# Impact of maternal micronutrient supplementation on pregnancy outcomes in developing countries: a systematic review and meta-analysis

**DOI:** 10.1186/s12884-026-09210-1

**Published:** 2026-05-13

**Authors:** Najia Sherzay, Siti Hamimah Sheikh Abdul Kadir, Noor Shafina Mohd Nor

**Affiliations:** 1 Cardiovascular Advancement and Research Excellence Institute (CARE Institute), Universiti Teknologi MARA (UiTM), Cawangan Selangor, Sungai Buloh, Selangor 47000 Malaysia; 2 Faculty of Medicine, Universiti Teknologi MARA (UiTM), Sungai Buloh, Selangor 47000 Malaysia; 3 Protein Production and Structure Core Facility, École polytechnique fédérale de Lausanne (EPFL), Lausanne, Switzerland

**Keywords:** Multiple micronutrients, poor pregnancy outcomes, Low birth weight, Small for gestational age

## Abstract

**Background:**

Poor pregnancy outcomes are still relatively high in developing countries. Deficiency in micronutrients is among the factors that play a role in developing adverse pregnancy outcomes. The present study aims to investigate the role of micronutrient deficiencies in poor pregnancy outcomes and their association with newborn health.

**Method:**

For this review, a systematic search was done using the Harzing Perish and publish software, as well as separate searches in PubMed, Google Scholar, Web of Science, and Scopus for evidence on the association between maternal micronutrient status and/or micronutrient supplementation during pregnancy and adverse pregnancy outcomes, including low birth weight (LBW) and small for gestational age (SGA). We assessed heterogeneity using the I2 index, conducted the meta-analysis, and measured the overall effect size using the fixed-effect method.

**Results:**

Overall, 49 articles were included in this systematic review, and 43 articles were included in the meta-analysis. Cumulative analysis of effect size showed that supplementation of vitamin D (d = 0.18), zinc (d = 0.39), iron with folic acid (d = 0.64) or multiple micronutrients (d = 0.059) plays positive role in reducing the risk of LBW and SGA. However, in all studies apart from iron with folic acid the effect of Multiple Micronutrient Supplementations (MMS) or other single micronutrient was assessed against the control group receiving iron with folic acid.

**Conclusion:**

Iron with folic acid showed the strongest and most consistent effect in reducing LBW and SGA; however, zinc, vitamin D, and multiple micronutrient supplementation were also associated with modest improvements in fetal growth outcomes.

The topic and protocol were retrospectively registered in PROSPERO (ID: CRD42023451468).

**Supplementary Information:**

The online version contains supplementary material available at 10.1186/s12884-026-09210-1.

## Introduction

Maternal diet and nutritional status play a major role in fetal health and growth [1]. The birth weight of an infant is a reliable index of intrauterine growth and a major factor determining child survival, future physical growth, and mental development. Birth weight also reflects the quality of life, socio-economic status, health awareness, and nutritional status of a community. The availability of food, energy, and proteins for pregnant women is always recommended, but the availability of micronutrients for a healthy pregnancy is also important [2]. In utero impact maternal undernutrition and micronutrient deficiencies has been linked to a higher risk of cardiovascular problems in adulthood, including a greater chance of high blood pressure, heart attack, stroke, heart failure, and type 2 diabetes [3]. There is growing evidence that has proven the importance of specific micronutrients for the specific time of pregnancy or for the prevention of specific postnatal disorders [4].

Zinc is one of the most important elements, which is present in all body fluids and tissues [5]. It has many important roles, including signal transduction, gene expression, cell proliferation, apoptosis, and the development of the neurological system during infancy [6]. It plays a role in many proteins and enzymes function; hence, its involvement in anabolic processes, including nucleic acid metabolism and protein synthesis [7]. During pregnancy and infancy, zinc plays a crucial role, particularly during embryogenesis, where its level in the fetus depends on maternal Zinc supplies [8]. According to the World Health Organization (WHO), the zinc requirement during pregnancy is 1.1 to 2 mg/day at early pregnancy, and the demand increases to 3 mg/day at later stages of pregnancy [9]. In early pregnancy, zinc is required for cell multiplication and differentiation, as well as organ formation in the fetus. During the early stages of pregnancy, a zinc deficiency can hinder the progress of the cell cycle, disrupt intracellular signalling, cause malfunctions in Zinc-related enzymes, and potentially lead to chromosomal damage [10].

Vitamin D is an important micronutrient during pregnancy, and maternal vitamin D deficiency has been associated with adverse outcomes including fetal growth restriction, placental dysfunction, pre-eclampsia, preterm birth, and low birth weight [11]. Furthermore, vitamin D deficiency during pregnancy is associated with an increased risk of long-term health consequences for the offspring, for example, asthma, multiple sclerosis, schizophrenia, type 1 diabetes, and autism [11]. Given that the placenta transfers only 60 to 80% of the mother’s vitamin D to the fetus, it’s crucial for the mother’s vitamin D level to surpass the standard 50 nmol/L, ideally reaching 75 nmol/L to guarantee a 50 nm/L fetal serum vitamin D level [12,13]. A common cause of anemia during pregnancy is iron and folic acid deficiency, which also leads to poor pregnancy outcomes [14]. Pregnancy increases the demand for iron and folic acid to meet the growing fetus’s requirements. Iron deficiency increases the risk of premature rupture of membranes (PROM), haemorrhage, and even maternal death [15]. Folic acid deficiency causes neural tube defects (NTD), preeclampsia, preterm delivery, birth anomalies, and type 2 diabetes later in life [16].

Poor pregnancy outcomes remain a major public health concern in developing countries. The World Health Organization (WHO) and maternal health researchers continue to investigate the underlying causes to reduce their incidence and improve neonatal survival. In this systematic review meta-analysis, our goal was to assess the effectiveness of multiple micronutrient supplementation during pregnancy in reducing the incidence of adverse outcomes, particularly low birth weight (LBW) and small for gestational age (SGA), within resource-limited settings.

## Methods

### Search strategy and screening

The review was conducted and reported in accordance with the Preferred Reporting Items for Systematic Reviews and Meta-Analyses (PRISMA) guidelines [17]. The topic and protocol were registered in PROSPERO (ID: CRD42023451468). Because the initial screening and selection process had already begun before registration, the protocol was registered retrospectively; however, the review followed a predefined methodological plan from the outset, minimizing the risk of selective reporting. A systematic literature search was conducted using Harzing’s Publish or Perish software (PubMed, Google Scholar, Scopus, Web of Science, Crossref) and individually within PubMed, Google Scholar, Scopus, and Web of Science. Search terms included controlled vocabulary (e.g., MeSH) and keywords related to micronutrients, pregnancy outcomes (LBW, SGA), and developing countries. The full search strategy for each database, including complete search strings and country filters, is provided in the supplemental information (Supplement Table 1 to Supplement Table 4).

We conducted the primary database search in June 2024 and updated it in in January 2026 to identify any new studies published. Two additional studies were identified [18,19] through this updated search these studies were reviewed for eligibility; however, they were not included in the quantitative synthesis. Although two additional studies published after the original search period were identified, they were not included in the meta-analysis due to methodological heterogeneity. One study assessed overall maternal dietary patterns rather than specific micronutrient supplementation or deficiency, while the other focused on dietary diversity and gestational weight gain as predictors of birth weight rather than isolated micronutrient exposures. Inclusion of these studies would have compromised the comparability required for quantitative synthesis. Nevertheless, their findings are consistent with the overall conclusions of this review, supporting the importance of maternal nutrition for optimal birth outcomes.

For the purposes of this review, ‘developing countries’ were defined according to the World Bank classification of low- and middle-income countries (LMICs). Countries included in the search strategy were also selected based on the World Bank LMIC classification.

### Data extraction

Screening of titles, abstracts, and full texts was conducted independently by two reviewers. EndNote was used for duplicate removal, and Microsoft Excel was used to organise the screening workflow, track eligibility decisions, and document reasons for exclusion. Disagreements were resolved by a third reviewer. Articles were retrieved and studied, all cross-sectional, case-control, cohort and randomized control trials conducted in any developing country with appropriate data of the effect of one or multiple micronutrients on pregnancy outcomes were included. Data was extracted by two authors independently and checked by a third author. The following information was extracted: first author, region of study, study population, type of micronutrient, and effect on neonatal outcome. Randomized controlled trials, cohort studies, case–control studies, and cross-sectional studies were included. Reviews, study protocols, editorials, conference abstracts without full data, case reports, animal studies, and other non-original research were excluded. The review primarily focused on studies conducted in low- and middle-income countries (LMICs); however, studies conducted in high-income countries were also included if the study populations consisted predominantly of immigrants originating from LMICs, as these populations share similar nutritional risk profiles and contextual determinants relevant to the review objectives. Studies were excluded only if the primary exposure of interest was maternal smoking, alcohol consumption, or specific environmental or occupational toxic exposures (e.g., heavy metals, pesticides, or industrial pollutants) as the main determinant of pregnancy outcomes. Studies in which smoking or alcohol use occurred among participants but was not the primary exposure and adjusted as a confounder were not excluded.

### Quality assessment

The quality of the included articles was assessed using the Strengthening the Reporting of Observational Studies in Epidemiology (STROBE) checklist. Two reviewers assessed the quality of studies independently, and a third reviewer double-checked the result. The included studies were with a minimum of 15 and a maximum of 22 points. The score of each article is written in the summary tables of each micronutrient.

### Risk of bias assessment

Study quality and risk of bias were assessed according to study design. Randomized controlled trials (RCTs) were evaluated using the Cochrane Risk of Bias tool version 2 (ROB-2), which assesses bias arising from the randomization process, deviations from intended interventions, missing outcome data, outcome measurement, and selective reporting. Non-randomized and observational studies (including cohort, case–control, and cross-sectional studies) were assessed using the Risk of bias in non-randomized studies of interventions (ROBINS-I) tool, covering confounding, participant selection, exposure classification, deviations from intended interventions, missing data, outcome measurement, and selective reporting.

### Certainty of evidence

The GRADE approach was used to assess the certainty of evidence. Observational studies began at low certainty but were upgraded to moderate certainty due to consistency of findings, biological plausibility, and acceptable precision. Randomized trials evaluating MMS achieved moderate certainty after minimal downgrading.

### Statistical analysis

Data were synthesized using a combination of narrative synthesis and quantitative meta-analysis. The term “conventional synthesis method” refers to the narrative integration of findings across studies when differences in design, outcome definitions, or effect measures did not allow statistical pooling. For quantitative synthesis, effect sizes were calculated separately for each study based on the reported outcome measures. Meta-analysis was conducted using IBM SPSS Statistics 29.0.2.0, applying the restricted maximum likelihood (REML) estimator within a random-effects model to account for between-study variability. Pooled effect sizes with 95% confidence intervals were generated, and results were presented in forest plots to illustrate the association of each micronutrient with LBW or SGA. Publication bias was assessed using Egger’s regression-based test, which evaluates small-study effects and asymmetry in funnel plots. Research integrity considerations (including during the screening process, the duplication, the retraction status, and the plausibility of data were reviewed; however, no studies were excluded based on integrity concerns. The analysis was reported separately for four nutrient groups: zinc, vitamin D, iron with folic acid, and multiple micronutrient supplementation.

## Results

The initial search identified 3,498 articles, of which 1,858 remained after duplicate removal using EndNote software. Following title and abstract screening, 1,327 studies were excluded, a total of 518 articles were assessed for eligibility, after 13 full-text reports could not be retrieved, after applying the inclusion and exclusion criteria, 43 studies were retained for meta-analysis and 49 for systematic review.

**Fig. 1:**
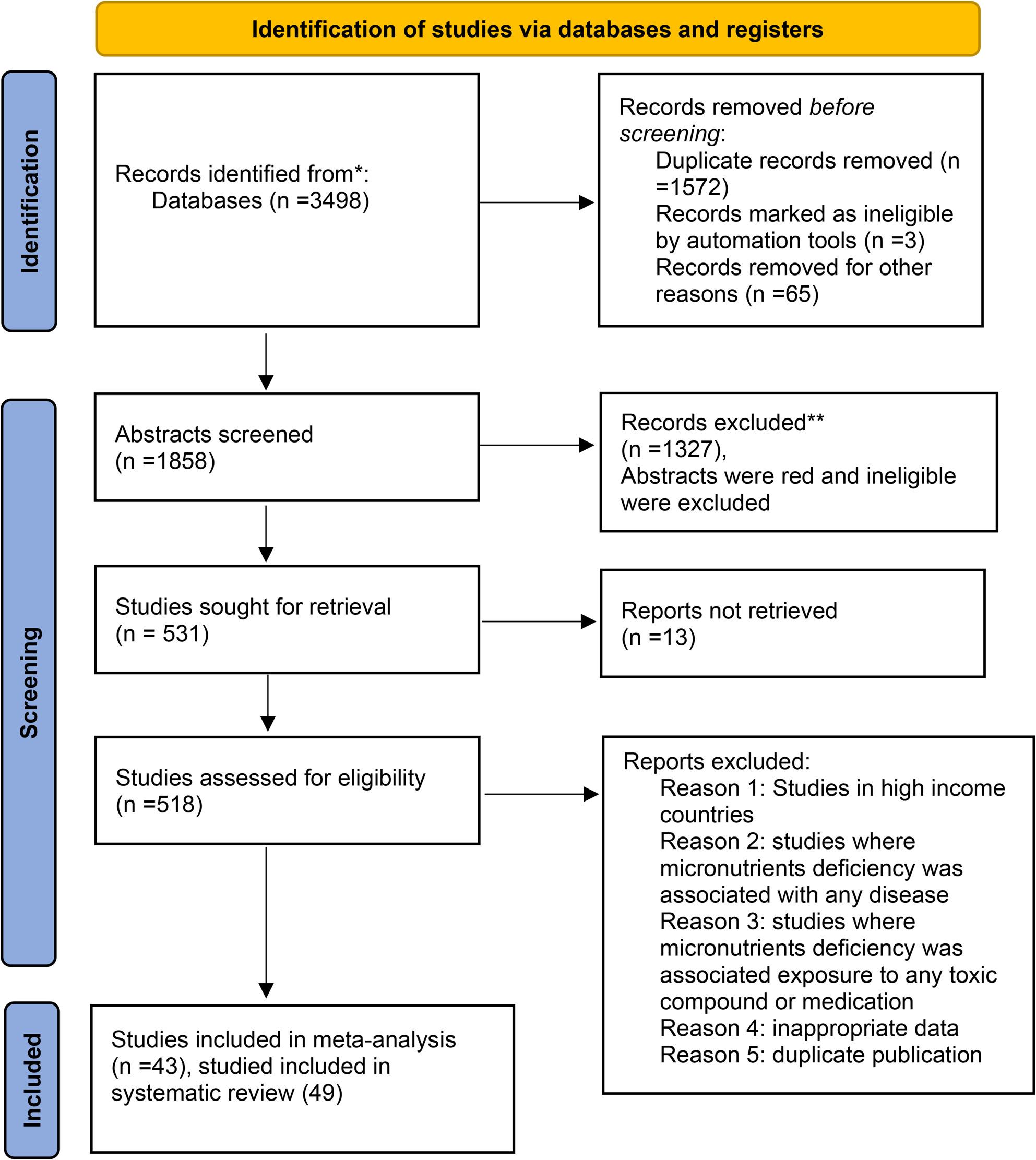
PRISMA flow diagram illustrating the study selection process for the systematic review and meta-analysis

A total of 49 unique studies were included in the systematic review. Of these, 12 studies assessed zinc, 18 assessed vitamin D, 10 assessed iron with folic acid, and 9 assessed multiple micronutrient supplementation (MMS). One study evaluated more than one micronutrient category and was therefore included in multiple subgroup analyses, resulting in a total count across categories exceeding the number of unique studies. The PRISMA flow diagram outlining the identification, screening, and eligibility process is presented in Fig. 1.

Most included studies were conducted in developing countries. The highest number of studies were from India (*n* = 7) and Iran (*n* = 7), followed by Pakistan (*n* = 6) and Iraq (*n* = 5). In addition, studies were conducted in Bangladesh (*n* = 3), Egypt (*n* = 2), Nigeria (*n* = 2), Morocco (*n* = 2), Tunisia (*n* = 2), Tanzania (*n* = 2), Nepal (*n* = 2), and Palestine/Gaza (*n* = 2). A small number of studies from higher-income settings, including Greece (*n* = 1) and Turkey (*n* = 1), were also included when the study populations consisted of immigrants originating from low- and middle-income countries. Single studies were additionally conducted in Kenya (*n* = 1), Benin (*n* = 1), South Africa (*n* = 1), Indonesia (*n* = 1), and Guinea-Bissau (*n* = 1).

Study designs varied and included cross-sectional, cohort, case–control, and randomized controlled trials, with sample sizes ranging from 80 to 31,290 participants.

The primary outcomes were low birth weight (LBW) and small for gestational age (SGA). Interventions and exposures evaluated included maternal supplementation or baseline status of Zinc, vitamin D, iron + folic acid, and multiple micronutrient combinations.

Risk of bias was assessed according to study design. Among the included studies, the majority of observational studies (*n* ≈ 39) were judged to have an overall moderate risk of bias based on ROBINS-I, primarily due to residual confounding, limited adjustment for socioeconomic and dietary factors, and variability in exposure assessment methods. Randomized controlled trials (*n* ≈ 10) were assessed using the Cochrane ROB-2 tool. Of these, most trials (*n* ≈ 8) were judged to have a low risk of bias, while a small number (*n* ≈ 2) presented *some concerns*, mainly related to deviations from intended interventions or incomplete outcome reporting. No randomized trial was judged to be at high risk of bias. Overall, the risk-of-bias assessment indicates that the available evidence is predominantly of moderate methodological quality, with randomized trials strengthening the certainty of evidence for multiple micronutrient supplementation outcomes.

Based on the GRADE evaluation, the overall certainty of evidence across all nutrient categories was moderate, supported by consistent findings across diverse populations, biologically plausible mechanisms linking micronutrient status to fetal growth, and acceptable precision of effect estimates. A minority of studies were downgraded to low certainty due to methodological limitations or inconsistency in effect direction.

### Effect of maternal Zinc level on pregnancy outcomes

**Fig. 2:**
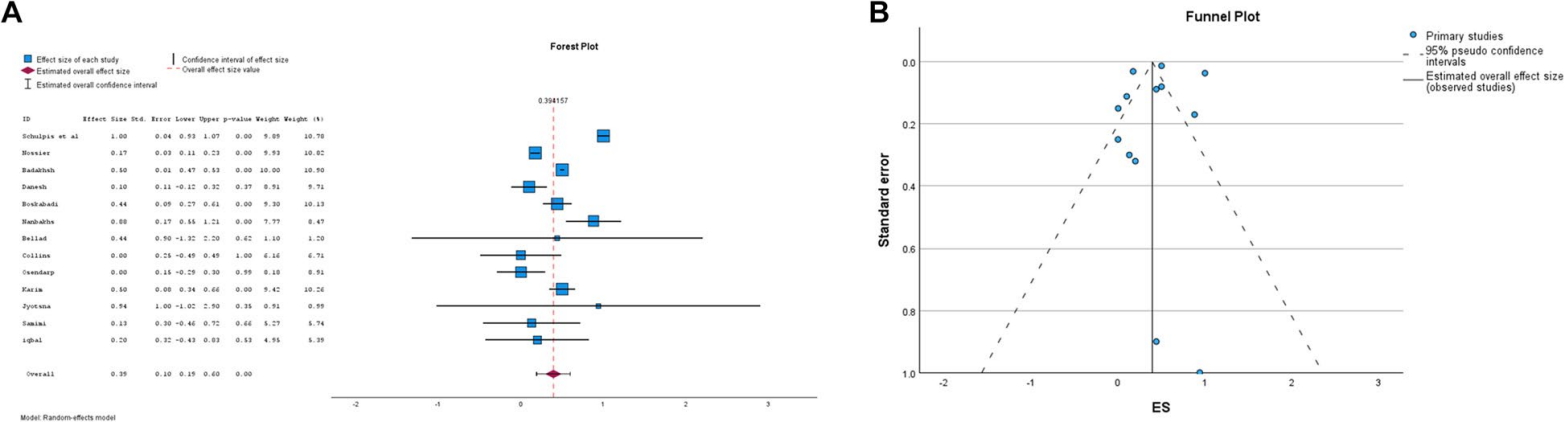
**(A)** Forest plot showing the standardized mean difference (SMD) and 95% confidence intervals for the effect of maternal Zinc levels on neonatal birth weight (in grams) across individual studies conducted in low- and lower-middle-income countries. The diamond indicates the pooled effect size, and the squares represent effect sizes of individual studies, with square size proportional to study weight. **B** Funnel plot assessing publication bias in studies reported the effect of maternal Zinc levels in neonatal birth weight. The vertical line represents the pooled effect size, and the dotted lines indicate pseudo 95% confidence limits

A total of 12 studies evaluated the association between maternal zinc status or zinc supplementation and pregnancy outcomes. Study designs included cross-sectional studies (*n* = 6), case–control studies (*n* = 3), prospective observational studies (*n* = 1), and randomized controlled trials (*n* = 2). Sample sizes ranged from 80 to 675 participants. Most studies assessed maternal or cord blood zinc concentrations, while a smaller number evaluated zinc supplementation during pregnancy. Overall, the majority of studies reported lower maternal zinc levels among mothers of low birth weight or small-for-gestational-age infants. The pooled effect size using a random-effects model showed that Zinc supplementation during pregnancy significantly reduces the risk of low birth weight (LBW) and small for gestational age (SGA). The standardized mean difference (SMD) was 0.39 (95% CI: 0.12 to 0.66, *p* < 0.05), with high heterogeneity (I² = 96%, *p* < 0.01). Egger’s test indicated no significant publication bias (*p* = 0.88) (Fig. 2).

A meta-regression was conducted to investigate potential sources of heterogeneity. Substantial between-study variability was confirmed (Q = 116.98, df = 12, *p* < 0.001; I² = 91%). Subgroup analysis based on the type of Zinc indicator (“Zinc Supplemented” vs. “Maternal Zinc Level”) revealed no significant between-group heterogeneity (Q = 0.50, df = 1, *p* = 0.48). Most studies reported that LBW newborns had lower levels of zinc in their mothers or cords and that there was a positive link between zinc intake and birth weight (Table 1). However, some studies from Iran and Nigeria did not find this link. Overall, findings support the small but meaningful effect of zinc adequacy on fetal growth.

**Table 1:** Association of maternal zinc (zn) deficiency with low birth weight in developing countries

Reference	Author	Country	Type of study	Number of participants	Study population / sample	Conclusion	STROBE points	Overall ROBINS-I	GRADE certainty
[21]	Schulpis et al.	Greece	Case-Control	499	Cord blood of newborns	Zn deficiency associated with LBW	19	Moderate	Moderate
[43]	Nossier et al.	Egypt	Randomized double-blind trial	675	Zn-deficient pregnant women	Zn supplementation has positive effect	20	Low	Moderate
[22]	Jyotsna et al.	India	Prospective	100	Mother–newborn pairs	Zn level lower in LBW babies and mothers	14	Moderate	Moderate
[24]	Samimi et al.	Iran	Case-Control	129	Mother–newborn pairs	No association with LBW	17	Moderate	Moderate
[44]	Badakhsh et al.	Iran	Cross-sectional	140	Mother–newborn pairs	Zn deficiency associated with LBW	18	Moderate	Moderate
[45]	Danesh et al.	Iran	Randomized double-blind trial	110	Mother–newborn pairs	Not reported	—	Low	Moderate
[8]	Iqbal et al.	Pakistan	Cross-sectional	80	Pregnant vs. non-pregnant women	Lower Zn associated with complications	19	Moderate	Low[
[46]	Nanbakhsh & Tabrizi	Iran	Cross-sectional	127	Mother–newborn pairs	Maternal Zn lower in LBW cases	18	Moderate	Moderate[
[47]	Bellad & K.S.	India	Cross-sectional	200	Mother–newborn pairs	Positive correlation maternal Zn ↔ birth weight	18	Moderate	Moderate
[48]	Collins	Nigeria	Cross-sectional	190	Mother–newborn pairs	No significant association	18	Moderate	Moderate
[49]	Osendarp et al.	Bangladesh	Case-control	559	Mother–newborn pairs	No difference Zn supplementation vs. placebo	17	Moderate	Moderate[
[50]	Karim et al.	Pakistan	Cross-sectional	382	Mother–newborn pairs	Maternal Zn improves neonatal BW	17	Moderate	Moderate

### Effect of maternal vitamin D level on low birth weight

**Fig. 3:**
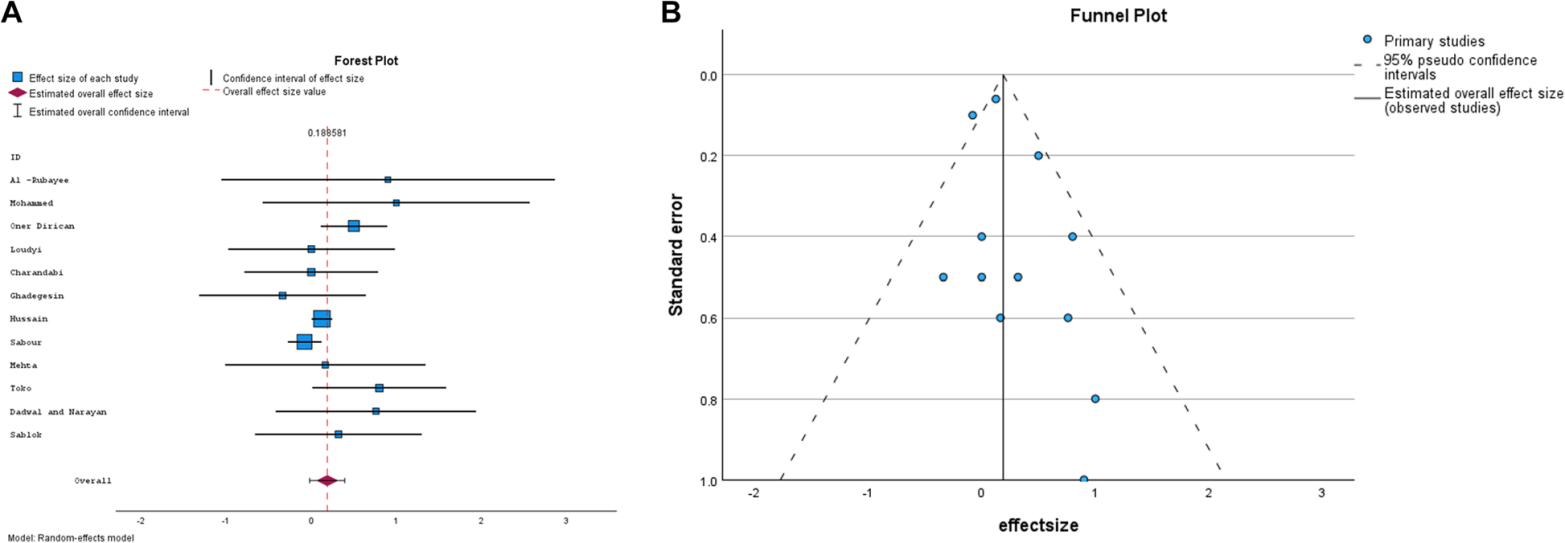
**A** Forest plot showing the standardized mean differences (SMDs) with 95% confidence intervals for the effect of maternal vitamin D levels on low birth weight (LBW) among neonates in developing countries. Each square represents an individual study's effect estimate, with square size reflecting study weight. The diamond at the bottom indicates the pooled overall effect size using a random-effects model. **B** Funnel plot used to evaluate publication bias for studies assessing the association between maternal vitamin D levels and LBW. The x-axis displays effect size (SMD), and the y-axis shows the standard error of each study. The vertical line marks the overall pooled estimate; dashed lines represent pseudo 95% confidence intervals

A total of 18 studies examined maternal vitamin D status or supplementation in relation to pregnancy outcomes. The majority of studies were cross-sectional (*n* = 10) or case–control (*n* = 6) in design, with two randomized or interventional studies. Sample sizes varied widely, ranging from 42 to 2,688 participants. Most studies reported a high prevalence of maternal vitamin D deficiency, while associations with low birth weight or preterm birth were inconsistent, likely reflecting heterogeneity in deficiency cut-offs, sunlight exposure, and supplementation regimens.The random-effects model indicated a small but positive effect of maternal vitamin D levels on reducing the risk of LBW. The pooled effect size was SMD = 0.19 (95% CI: 0.01 to 0.37, *p* < 0.05), with moderate heterogeneity (I² = 43%). Egger’s test showed no evidence of publication bias (*p* = 0.79) (Fig. 3). (Table 2).

**Table 2:** Effect of maternal vitamin D deficiency on low birth weight in developing countries

Reference	Author	Country	Type of study	Sample Size	Results and Consequences	STROBE points	Overall ROBINS-I	GRADE certainty
[27]	Ahmed F et al.	Bangladesh	Cross-sectional	515	17.3% of women deficient; association with pregnancy outcome not reported	18	Moderate	Moderate
[51]	Salek M et al.	Iran	Cross-sectional	88	Higher percentage of vitamin D deficiency	16	Moderate	Moderate
[52]	Hantoosh HA et al.	Iraq	Cross-sectional	500	76% deficient, 18% insufficient, 7% sufficient	15	Moderate	Moderate
[28]	Kasim SF et al.	Iraq	Case-control	80	95% of women with spontaneous abortion had vitamin D deficiency	15	Moderate	Moderate
[29]	Al-Rubaye WEL et al.	Iraq	Cross-sectional	88	96% deficient; maternal level positively correlated to neonatal vitamin D and birth weight	18	Moderate	Moderate
[30]	Mohammed AK et al.	Iraq	Cross-sectional	42	Vitamin D deficiency significantly associated with early pregnancy loss	15	Moderate	Moderate
[53]	Oner Dirican A et al.	Turkey	Retrospective	2688	Lower preterm birth and LBW rates in vitamin D supplemented group vs. non-supplemented	19	Moderate	Moderate
[54]	Mwafy SN et al.	Gaza	Case-control	200	Deficiency significantly higher in pregnant women vs. non-pregnant controls	15	Moderate	Moderate
[55]	Loudyi FM et al.	Morocco	Cross-sectional	102	90.1% of pregnant women deficient; deficiency not associated with neonatal birth weight	16	Moderate	Moderate
[56]	Nasri K et al.	Tunisia	Case-control	132	Vitamin D deficiency associated with increased risk of neural tube defects	18	Moderate	Moderate
[57]	Charandabi SMA et al.	Iran (Tabriz)	Case-control	126	No effect of vitamin D supplementation on mode of delivery or neonatal birth weight	17	Moderate	Moderate
[31]	Gbadegesin A et al.	Nigeria	Cross-sectional	461	No differences in pregnancy complications or caesarean rate between vitamin D groups	16	Moderate	Moderate
[58]	Hossain N et al.	Pakistan	Case-control	200	Supplementation improved neonatal vitamin D and 5-min Apgar; no significant effect on anthropometrics	18	Moderate	Moderate
[59]	Sabour H et al.	Iran	Cross-sectional	449	Vitamin D supplementation correlated with higher 1-min Apgar and birth weight	16	Moderate	Moderate
[60]	Mehta S et al.	Tanzania	Cross-sectional	884	Deficiency not associated with LBW or poor outcomes; 2-fold higher mother-to-child HIV transmission	15	Moderate	Moderate
[61]	Toko EN et al.	Kenya	Cross-sectional	63	Deficient plasma vitamin D level associated with 4-fold higher risk of stunting	19	Moderate	Moderate
[62]	Dadwal M et al.	India	Case-control	100	Maternal deficiency associated with higher risk of preterm birth	15	Moderate	Moderate
[63]	Sablok A et al.	India	Case-control	165	Vitamin D supplementation associated with lower risk of SGA	16	Moderate	Moderate

Most studies reported extremely high rates of vitamin D deficiency in LMICs (> 70% in several populations) (Table 3). Some supplementation trials improved neonatal vitamin D status, Apgar scores, and modestly increased birth weight. However, multiple cross-sectional studies detected no association with LBW, likely due to varying deficiency cut-offs, sunlight exposure, and assay variability.

**Table 3:** Effect of maternal anaemia and iron deficiency on low birth weight in developing countries

Reference	Author	Country	Type of study	Sample Size	Results and Consequences	STROBE points	Overall ROBINS-I	GRADE certainty
[32]	El-Farrash R et al.	Egypt	Case-control	80	Neonates of anaemic mothers had significantly lower birth weight than those of non-anaemic mothers	16	Moderate	Moderate
[33]	Finkelstein JL et al.	India	Cross-sectional	366	Anaemia associated with ~ 2-fold higher risk of LBW	18	Moderate	Moderate
[64]	Bodeau-Livinec F et al.	Benin	Cross-sectional	1508	Higher prevalence of LBW in severely anaemic women (prevalence ratio 2.8, 95% CI)	18	Moderate	Moderate
[65]	Mahmood T et al.	Pakistan	Cross-sectional	622	Third-trimester anaemia associated with adverse maternal and neonatal outcomes	18	Moderate	Moderate
[66]	Nair M et al.	India	Cohort	1007	Iron deficiency anaemia associated with higher risk of LBW, SGA and perinatal deaths	16	Moderate	Moderate
[67]	Mahato V et al.	Nepal	Cross-sectional	200	Mild/moderate anaemia linked with common complications; severe anaemia linked with IUGR, LBW, perinatal death	17	Moderate	Moderate
[68]	Tunkyi K et al.	South Africa	Cross-sectional	2000	Stillbirth significantly higher in anaemic group; LBW higher but not significant	17	Moderate	Moderate
[69]	Srour MA et al.	Palestine	Cross-sectional	163	Maternal ferritin significantly associated with LBW and preterm delivery	19	Moderate	Moderate
[70]	Suprapto TCM et al.	Iraq	Cross-sectional	500	Iron deficiency anaemia associated with increased risk of poor birth outcomes (LBW, preterm, adverse maternal outcomes)		Moderate	Moderate
[35]	Bakhtiar UJ et al.	Pakistan	Cross-sectional	860	Maternal anaemia associated with LBW, preterm birth and intrauterine foetal death	16	Moderate	Moderate

### Effect of maternal folic acid + iron level on low birth weight

**Fig. 4:**
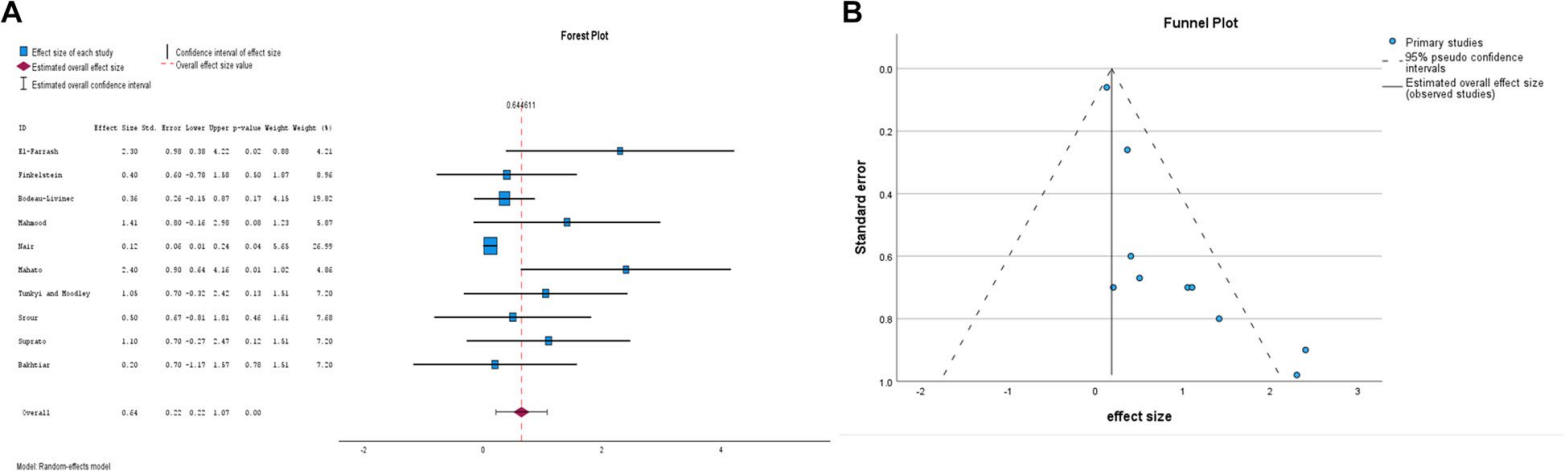
**A** Forest plot illustrating the standardized mean differences (SMDs) and 95% confidence intervals for the effect of maternal iron and folic acid supplementation on the risk of low birth weight (LBW) in newborns across studies conducted in developing countries. Each square represents an individual study’s effect size, with the square size proportional to its weight. The diamond at the bottom represents the pooled effect estimate using a random-effects model. **B** A funnel plot assesses publication bias in the studies included. The x-axis indicates the effect size (SMD), while the y-axis shows the standard error. The vertical line shows the pooled effect size, and the dashed lines represent the pseudo 95% confidence limits

A total of 10 studies assessed maternal anaemia or iron–folic acid status in relation to pregnancy outcomes. Study designs included cross-sectional studies (*n* = 7), case–control studies (*n* = 1), and cohort studies (*n* = 2), with sample sizes ranging from 80 to 2,000 participants. Most studies consistently reported higher risks of low birth weight, small for gestational age, stillbirth, or preterm birth among anaemic mothers, particularly in cases of moderate-to-severe anaemia during late pregnancy. The pooled effect size for maternal iron plus folic acid supplementation on LBW was SMD = 0.64 (95% CI: 0.25 to 1.03, *p* < 0.01), suggesting a significant positive impact. Heterogeneity was moderate (I² = 51.8%), and no publication bias was detected (Egger’s test: *p* = 0.63) (Fig. 4).

Across studies, anaemia—particularly moderate-to-severe anaemia in late pregnancy—was consistently associated with LBW, SGA, stillbirth, and adverse maternal outcomes. The findings correspond with the well-established biological roles of iron and folate in oxygen transport and early fetal neural development (Table 4).

**Table 4:** Effect of maternal multiple micronutrients (MMNs) supplementation on low birth weight in developing countries

Reference	Author	Country	Type of study	Sample Size	Results and Consequences	STROBE points	Overall ROBINS-I	GRADE certainty
[36]	Elfane H et al.	Morocco	Cross-sectional case-control	344	Higher MMN intake in mothers of normal-weight babies vs. LBW	17	Moderate	Moderate
[38]	Fares S et al.	Tunisia	Case-control	907	Micronutrient deficiency significantly associated with LBW	17	Moderate	Moderate
[71]	West KP et al.	Bangladesh	Cluster randomized double-masked trial	25,130	MMN supplementation reduced preterm births and LBW vs. iron–folic acid	18	Low	Moderate
[72]	Christian et al.	Nepal	Case-control		MMN use associated with reduced LBW and preterm birth vs. control	19	Moderate	Moderate
[40]	Tukadoji R et al.	India	Cross-sectional		Positive correlation between maternal MMN intake and neonatal birth weight	14	Moderate	Low
[41]	Bhutta ZA et al.	Pakistan	Case-control	2378	MMS significantly decreased LBW vs. control; non-significant reduction vs. iron–folic acid	17	Moderate	Moderate
[42]	Shankar A et al.	Indonesia	Double-blind randomized controlled trial	31,290	18% reduction in adverse outcomes in MMS group vs. iron–folic acid	18	Low	Moderate
[73]	Fawzi WW et al.	Tanzania	Double-blind trial	8428	MMN supplementation reduced LBW and SGA, but not prematurity or foetal death	17	Low	Moderate
[37]	Kæstel P et al.	Guinea-Bissau	Randomized double-masked trial	2100	MMN increased birth weight but did not reduce perinatal mortality	19	Low	Moderate

### Effect of multiple micronutrients on reducing low birth weight

**Fig. 5:**
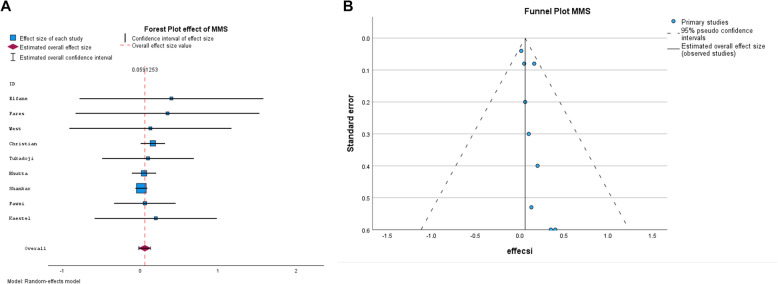
**A** Forest plot showing the standardized mean differences (SMDs) and 95% confidence intervals for the effect of multiple micronutrient supplementation (compared to iron + folic acid) during pregnancy on the risk of low birth weight (LBW) in developing countries. Each square represents the effect size of an individual study, with size proportional to study weight. The diamond represents the pooled effect size using a random-effects model. **B** Funnel plot for assessing publication bias across the included studies. The x-axis represents the effect size (SMD), and the y-axis shows the standard error. The vertical line indicates the overall effect size, and the dashed lines show the 95% pseudo confidence limits

A total of 9 studies evaluated the effects of multiple micronutrient supplementation during pregnancy. Study designs included large randomized controlled trials (*n* = 5), case–control studies (*n* = 2), and cross-sectional studies (*n* = 2). Sample sizes ranged from 344 to over 31,000 participants. Most randomized trials compared multiple micronutrient (MMN) with iron–folic acid supplementation and reported modest reductions in low birth weight and small-for-gestational-age outcomes, although differences were sometimes not statistically significant. In studies comparing multiple micronutrient (MMN) supplementation to iron with folic acid alone, the overall effect size was SMD = 0.05 (95% CI: − 0.10 to 0.20, *p* > 0.05), suggesting no significant difference. Heterogeneity was low (I² = 7.9%), and Egger’s test showed no evidence of publication bias (*p* = 0.79) (Fig. 5).

Narrative evidence indicates that large-scale RCTs from Bangladesh, Indonesia, and Tanzania consistently showed reductions in LBW and SGA with MMN supplementation, though differences compared with IFA alone were sometimes modest or statistically non-significant (Table 4). The combined biological role of multiple micronutrients supports improved foetal growth, especially in populations with widespread deficiencies.

## Discussion

This systematic review and meta-analysis evaluated the association between maternal micronutrient status—including Zinc, vitamin D, iron deficiency anaemia, and multiple micronutrient supplementation and the risk of adverse neonatal outcomes such as low birth weight (LBW) and small for gestational age (SGA). Across the included studies, the findings consistently suggest that inadequate maternal micronutrient levels negatively influence fetal growth, although the strength and consistency of associations vary across nutrients and study designs.

A critical appraisal of the included studies indicates that the overall quality of evidence was modest, most observational studies showed a moderate risk of bias according to ROBINS-I, while randomized controlled trials generally demonstrated a low risk of bias or some concerns based on ROB-2. Many primary studies lacked adequate adjustment for important confounders such as maternal diet, socioeconomic status, inflammation, comorbidities, and coexisting micronutrient deficiencies. These unmeasured factors may have influenced the reported associations between micronutrient status and neonatal outcomes. Additionally, heterogeneity in study design, population characteristics, sample size, timing of micronutrient assessment, and laboratory assay methods likely contributed to the variability of findings. Despite these limitations, the biological plausibility supporting the role of micronutrients in fetal growth is well established, which enhances confidence in the consistency of findings across diverse settings.

Contextual differences between low- and middle-income countries (LMICs) and high-income countries (HICs) may also influence the interpretation of these findings. The vast majority of included studies were conducted in LMICs, where baseline micronutrient deficiencies are common due to limited dietary diversity, low-quality diets, food insecurity, and gaps in antenatal supplementation coverage. In such settings, micronutrient deficiencies likely impose a greater physiological burden on fetal development, which may amplify the apparent benefits of supplementation. In contrast, the limited studies conducted in high-income countries predominantly focused on immigrant or socioeconomically disadvantaged populations rather than the general population, indicating a distinct nutritional and environmental context. Therefore, while the overall direction of the effect was consistent across studies, the magnitude of the benefit observed in LMICs may be greater due to higher baseline deficiency rates and lower access to fortified foods or structured supplementation programs. These contextual differences demonstrate the value of tailoring maternal nutrition policies to local resource availability and population needs.

Zinc plays a fundamental role in fetal development and cellular growth. Deficiency is a major contributor to morbidity and growth impairment, particularly stunting, in many low-resource settings [20,21]. In the present analysis of 12 zinc studies from developing countries, we observed a generally positive association between maternal zinc status and improved neonatal birth weight. Several studies have documented higher cordblood zinc levels and improved fetal growth among infants born to zinc-sufficient mothers. Randomized controlled trials also reported reductions in outcomes such as stillbirth, preterm birth, and early neonatal morbidity in zinc-supplemented groups, although not all trials demonstrated a significant increase in birth weight.

Nevertheless, considerable heterogeneity was noted across zinc studies. Sensitivity and subgroup analyses confirmed that the pooled effect remained stable after excluding high-risk studies and under leave-one-out testing, suggesting the robustness of the findings. Part of the observed heterogeneity may reflect differences in study sample sizes, baseline nutritional status, and inconsistent laboratory assays for zinc assessment. Importantly, some studies reported no significant association between zinc status and birth weight, underscoring variability in dietary patterns, zinc absorption, and measurement methods across populations [22,23]. Biological plausibility remains strong: zinc regulates IGF-1 activity in osteoblasts, supports placental alkaline phosphatase activity, and is required for DNA synthesis and cell proliferation—all critical for foetal skeletal and cellular growth [24].

The inconsistent associations observed between maternal vitamin D status and pregnancy outcomes may be explained by heterogeneity in study design, exposure assessment, and contextual factors such as sunlight exposure and baseline nutritional status. Although biological plausibility supports a role for), placental function [8], calcium regulation [25], and immune modulation [26]. Our systematic review showed a high prevalence of vitamin D deficiency across developing countries, reinforcing the need for supplementation strategies during pregnancy [27–30]. However, some studies showed no association between vitamin D status and select perinatal outcomes [31], reflecting variation in cut-off thresholds for deficiency, maternal sunlight exposure, and assay variability.

Iron deficiency anaemia, long recognised as a major public health concern among women of reproductive age, demonstrated a stronger and more consistent association with foetal growth outcomes [32,33]. In our analysis, iron–folic acid supplementation was significantly associated with increased birth weight and reduced risk of LBW. The link between folate deficiency and neural tube defects (NTDs) is well established [34]; thus, inadequate early-pregnancy folic acid intake continues to contribute to preventable congenital anomalies [35], particularly in regions where awareness and supplementation coverage remain low.

Multiple micronutrient supplementation typically containing iron, Zinc, calcium, vitamins D, B-complex, C, E, and other essential nutrients—showed beneficial effects on foetal development across several trials [36–38]. As these micronutrients play key roles in one-carbon metabolism, cell proliferation, and differentiation, their combined supplementation is biologically plausible in supporting optimal intrauterine growth [39]. During pregnancy and lactation, the foetus relies entirely on maternal nutrient stores, making maternal deficiencies particularly consequential for fetal development [40–42].

Across nutrient categories, the overall certainty of evidence using GRADE was moderate, supported by consistent direction of effects, strong biological plausibility, and acceptable precision. Most observational studies had a moderate risk of bias according to ROBINS-I largely due to residual confounding, limited adjustment for covariates, and variability in micronutrient assessment techniques. Given differences in study design, sample size, population characteristics, supplementation dosage and duration, and laboratory measurement techniques, we expect significant heterogeneity across studies.

## Limitations

This review has several important limitations. Most of the included studies were observational, which restricts causal inference and increases the likelihood of residual confounding. Many primary studies did not adequately adjust for key maternal factors such as socioeconomic status, dietary diversity, inflammation or infection, and coexisting micronutrient deficiencies, which may have influenced the observed associations.

There was also substantial heterogeneity across studies. Differences in study design, population characteristics, baseline nutritional status, sample sizes, supplement formulations, and the timing and dosage of interventions may have contributed to the variability in effect estimates. Geographic diversity and differing methodological quality across studies further complicate direct comparison.

Although funnel plot assessments suggested minimal asymmetry, the possibility of publication bias cannot be completely excluded, particularly in low- and middle-income countries where smaller studies with null findings may remain unpublished.

Finally, variation in micronutrient assessment methods—including differences in laboratory assays, biomarker thresholds, and whether nutrient status was evaluated through serum levels, supplementation records, or dietary intake—may have affected the accuracy and comparability of findings across studies.

## Conclusion

In summary, this meta-analysis and systematic review showed that the supplementation of micronutrients in developing countries has a positive effect on reducing the chances of an important poor pregnancy outcome of LBW and SGA in the neonates. The most efficient micronutrient supplementation was iron with folic acid. Furthermore, supplementation of zinc, vitamin D, or other multiple vitamins and minerals further helps reduce the chances of LBW cases in developing countries.

## Supplementary Information

Supplementary Material 1

Supplementary Material 2

Supplementary Material 3

Supplementary Material 4

## Data Availability

The data supporting the findings of this study are available from the corresponding author upon reasonable request.
