# Supplementary material for: Impact of maternal micronutrient supplementation on pregnancy outcomes in developing countries: a systematic review and meta-analysis
Source: BMC Pregnancy Childbirth. 2026 May 13;26:731. doi: 10.1186/s12884-026-09210-1 (PMC13343903; doi:10.1186/s12884-026-09210-1)
Supplement: Supplementary file 1 — Supplementary Material 1 [file 12884_2026_9210_MOESM1_ESM.docx]

Full Search Strategy

Supplementary File 1. Complete Search Strategy for All Databases

Databases Searched

By using Harzing’s Publish or Perish software searched in (PubMed, Scopus, Web of Science, Google Scholar, CrossRef)

Furthermore following databases were also searched individually

- PubMed

- Scopus

- Web of Science

- Google Scholar

Search Dates

- Initial search: June 2024

- Updated search: September 2024

Search Strategy

The search strategy combined medical subject headings (MeSH), keywords, and Boolean operators. No date restrictions were applied. Searches were limited to human studies, English language, and original research.

1. PubMed Search Strategy

(("micronutrient*" OR "trace element*" OR zinc OR "vitamin D"

OR "folic acid" OR iron OR "iron folic acid" OR

"multiple micronutrient*" OR MMN)

AND

("pregnancy outcome*" OR "poor pregnancy outcome*"

OR "low birth weight" OR LBW OR "small for gestational age" OR SGA

OR "preterm birth" OR stillbirth)

AND

("developing countr*" OR "low income countr*" OR "middle income countr*"

OR Pakistan OR India OR Nepal OR Bangladesh OR Iran OR Iraq OR Nigeria

OR Benin OR Tanzania OR Kenya OR Ethiopia OR Ghana OR Morocco OR Tunisia

OR Palestine OR Egypt))

2. Scopus Search Strategy

TITLE-ABS-KEY ( micronutrient* OR zinc OR "vitamin D" OR "folic acid"

OR iron OR "iron folic acid" OR "multiple micronutrient*" )

AND

TITLE-ABS-KEY ( "low birth weight" OR LBW OR "small for gestational age"

OR "pregnancy outcome*" )

AND

TITLE-ABS-KEY ( "developing countr*" OR Pakistan OR India OR Nepal

OR Bangladesh OR Iran OR Iraq OR Nigeria OR Egypt )

3. Web of Science Search Strategy

TS=(micronutrient* OR zinc OR "vitamin D" OR "folic acid"

OR iron OR "iron folic acid" OR "multiple micronutrient*")

AND

TS=("poor pregnancy outcome*" OR "low birth weight" OR LBW

OR "small for gestational age")

AND

TS=("developing countr*" OR Pakistan OR India OR Nepal OR Bangladesh

OR Iran OR Iraq OR Nigeria OR Egypt OR Tunisia OR Morocco)

4. Google Scholar (via Publish or Perish)

("micronutrient deficiency" OR "zinc deficiency"

OR "vitamin D deficiency" OR "folic acid"

OR "iron deficiency anaemia")

AND

("low birth weight" OR LBW OR "small for gestational age")

AND

("developing countries" OR Pakistan OR India OR Bangladesh

OR Iran OR Nigeria OR Morocco)

5. Additional Notes

- Manual reference list screening (“snowballing”) was performed.

- Grey literature, reviews, and conference abstracts were excluded.
