# Supplementary material for: Impact of maternal micronutrient supplementation on pregnancy outcomes in developing countries: a systematic review and meta-analysis
Source: BMC Pregnancy Childbirth. 2026 May 13;26:731. doi: 10.1186/s12884-026-09210-1 (PMC13343903; doi:10.1186/s12884-026-09210-1)
Supplement: Supplementary file 2 — Supplementary Material 2 [file 12884_2026_9210_MOESM2_ESM.docx]

Supplementary File 2

ROBINS-I Risk of Bias Summary

Risk of Bias Assessment of Included Studies Using the ROBINS-I Tool

**Overview**

Risk of bias was assessed for all non-randomized studies using the ROBINS-I tool, evaluating seven core domains:

- Confounding

- Selection of participants

- Classification of exposures

- Deviations from intended exposures

- Missing data

- Measurement of outcomes

- Selection of reported results

**Interpretation**

- No study was rated as critical risk of bias.

- Moderate risk was common due to limited control of confounders, non-standard laboratory methods, and cross-sectional design predominance.

- Large RCTs assessing MMS were generally low risk, strengthening conclusions for this category.

- Heterogeneity in exposure measurement contributed to variability in findings.

**A. Robin I Risk of Bias of studies included in assessment of Zn**

| Reference | Country | Study Type | Confounding | Selection | Exposure Classification | Deviations | Missing Data | Outcome Measurement | Reporting | Overall Risk |
| --- | --- | --- | --- | --- | --- | --- | --- | --- | --- | --- |
| ^14^ | Greece | Case-Control | Moderate | Moderate | Low | Low | Moderate | Low | Moderate | Moderate |
| ^36^ | India | Prospective | Moderate | Low | Low | Low | Moderate | Low | Low | Moderate |
| ^15^ | Iran | Case-Control | Moderate | Moderate | Low | Low | Low | Low | Moderate | Moderate |
| ^17^ | Iran | Cross-sectional | Serious | Moderate | Moderate | Low | Low | Low | Moderate | Serious |
| ^37^ | Pakistan | Cross-sectional | Serious | Moderate | Moderate | Low | Low | Low | Moderate | Serious |
| ^38^ | Iran | Cross-sectional | Serious | Low | Moderate | Low | Low | Low | Moderate | Serious |
| ^6^ | India | Cross-sectional | Serious | Low | Moderate | Low | Low | Low | Moderate | Serious |
| ^39^ | Nigeria | Cross-sectional | Serious | Moderate | Moderate | Low | Low | Low | Moderate | Serious |
| ^40^ | Bangladesh | Case-Control | Moderate | Moderate | Low | Low | Low | Low | Moderate | Moderate |
| ^41^ | Pakistan | Cross-sectional | Serious | Low | Moderate | Low | Low | Low | Moderate | Serious |

**B. Robin I Risk of Bias of studies included in assessment of vitamin D**

| Study | Bias due to confounding | Bias in selection of participants | Bias in classification of interventions/exposure | Bias due to deviations from intended interventions/exposure | Bias due to missing data | Bias in measurement of outcomes | Bias in selection of the reported result | Overall ROBINS-I risk of bias | GRADE certainty of evidence |
| --- | --- | --- | --- | --- | --- | --- | --- | --- | --- |
| ^20^ | Serious | Moderate | Low | Low | Moderate | Moderate | Low | Serious | Very low |
| ^44^ | Serious | Moderate | Low | Low | Moderate | Moderate | Low | Serious | Very low |
| ^45^ | Serious | Moderate | Low | Low | Moderate | Moderate | Low | Serious | Very low |
| ^21^ | Serious | Serious | Low | Low | Moderate | Moderate | Low | Serious | Very low |
| ^22^ | Serious | Moderate | Low | Low | Moderate | Low–Moderate | Low | Serious | Very low |
| ^23^ | Serious | Moderate | Low | Low | Serious | Moderate | Low | Serious | Very low |
| ^46^ | Serious | Moderate | Low | Low | Low | Low | Low | Serious | Low |
| ^47^ | Serious | Serious | Low | Low | Moderate | Moderate | Low | Serious | Very low |
| ^48^ | Serious | Moderate | Low | Low | Moderate | Moderate | Low | Serious | Very low |
| ^49^ | Serious | Serious | Low | Low | Moderate | Moderate | Low | Serious | Very low |
| ^50^ | Serious | Moderate–Serious | Low | Low | Moderate | Moderate | Low | Serious | Very low |
| ^24^ | Serious | Moderate | Low | Low | Moderate | Moderate | Low | Serious | Very low |
| ^51^ | Serious | Serious | Low | Low | Moderate | Moderate | Low | Serious | Very low |
| ^52^ | Serious | Moderate | Low | Low | Moderate | Moderate | Low | Serious | Very low |
| ^53^ | Serious | Moderate | Low | Low | Moderate | Moderate | Low | Serious | Very low |
| ^54^ | Serious | Moderate | Low | Low | Moderate | Moderate | Low | Serious | Very low |
| ^55^ | Serious | Serious | Low | Low | Moderate | Moderate | Low | Serious | Very low |
| ^56^ | Serious | Serious | Low | Low | Moderate | Moderate | Low | Serious | Very low |

**C. Robin I Risk of Bias of studies included in assessment of Iron and folic acid**

| Study | Bias due to confounding | Bias in selection of participants | Bias in classification of interventions/exposure | Bias due to deviations from intended interventions/exposure | Bias due to missing data | Bias in measurement of outcomes | Bias in selection of the reported result | Overall ROBINS-I risk of bias | GRADE certainty of evidence |
| --- | --- | --- | --- | --- | --- | --- | --- | --- | --- |
| ^25^ | Serious | Serious | Low | Low | Moderate | Moderate | Low | Serious | Very Low |
| ^26^ | Serious | Moderate | Low | Low | Moderate | Moderate | Low | Serious | Very Low |
| ^57^ | Serious | Moderate | Low | Low | Moderate | Moderate | Low | Serious | Very Low |
| ^58^ | Serious | Moderate | Low | Low | Moderate | Moderate | Low | Serious | Very Low |
| ^59^ | Moderate–Serious | Moderate | Low | Low | Moderate | Low–Moderate | Low | Serious | Low |
| ^60^ | Serious | Moderate | Low | Low | Moderate | Moderate | Low | Serious | Very Low |
| ^61^ | Serious | Moderate | Low | Low | Moderate | Moderate | Low | Serious | Very Low |
| ^62^ | Serious | Moderate | Low | Low | Moderate | Moderate | Low | Serious | Very Low |
| ^63^ | Serious | Moderate | Low | Low | Moderate | Moderate | Low | Serious | Very Low |
| ^28^ | Serious | Moderate | Low | Low | Moderate | Moderate | Low | Serious | Very Low |

**D. Robin I Risk of Bias of studies included in assessment of Multiple Micronutrients**

| Study | Bias due to confounding | Bias in selection of participants | Bias in classification of interventions/exposure | Bias due to deviations from intended interventions/exposure | Bias due to missing data | Bias in measurement of outcomes | Bias in selection of the reported result | Overall ROBINS-I risk of bias | GRADE certainty of evidence |
| --- | --- | --- | --- | --- | --- | --- | --- | --- | --- |
| ^29^ | Serious | Serious | Low | Low | Moderate | Moderate | Low | Serious | Very Low |
| ^31^ | Serious | Serious | Low | Low | Moderate | Moderate | Low | Serious | Very Low |
| ^64^ | Low | Low | Low | Low | Moderate | Low | Low | Moderate | Moderate |
| ^65^ | Serious | Moderate | Low | Low | Moderate | Moderate | Low | Serious | Low |
| ^33^ | Serious | Serious | Low | Low | Moderate | Moderate | Low | Serious | Very Low |
| ^34^ | Serious | Moderate | Low | Low | Moderate | Moderate | Low | Serious | Low |
| ^35^ | Low | Low | Low | Low | Moderate | Low | Low | Moderate | Moderate |
| ^66^ | Low | Low–Moderate | Low | Low | Moderate | Low | Low | Moderate | Moderate |
| ^30^ | Low | Low–Moderate | Low | Low | Moderate | Low | Low | Moderate | Moderate |
