# Supplementary material for: Impact of maternal micronutrient supplementation on pregnancy outcomes in developing countries: a systematic review and meta-analysis
Source: BMC Pregnancy Childbirth. 2026 May 13;26:731. doi: 10.1186/s12884-026-09210-1 (PMC13343903; doi:10.1186/s12884-026-09210-1)
Supplement: Supplementary file 3 — Supplementary Material 3 [file 12884_2026_9210_MOESM3_ESM.docx]

Supplementary File 3: GRADE Summary of Findings

**A: GRADE Table — Zinc (Zn)**

| Reference | GRADE Certainty | Reason for GRADE Rating |
| --- | --- | --- |
| 14 | Moderate | Observational design but adequate sample size; consistent findings; moderate risk of bias. |
| 36 | Moderate | Randomized trial but downgraded due to unclear reporting and some methodological limitations. |
| 15 | Moderate | Prospective observational study; moderate sample; some confounding likely. |
| 17 | Moderate | Case–control design; moderate risk of bias; outcome measurement acceptable. |
| 37 | Moderate | Observational design; acceptable sample size; moderate confounding risk. |
| 38 | Moderate | RCT but downgraded for incomplete outcome reporting and unclear risk-of-bias domains. |
| 6 | Low | Small sample size; substantial confounding; limited generalizability. |
| 39 | Moderate | Observational design with reasonable sample; moderate confounding; consistent results. |
| 40 | Moderate | Observational design; moderate risk of bias; adequate measurement but limited adjustment. |
| 41 | Moderate | Observational design; moderate methodological limitations; consistent measurement. |
| 42 | Moderate | Case–control design; moderate risk of bias; limited control for confounding. |
| 43 | Moderate | Observational design; acceptable sample; moderate residual confounding. |

**B. GRADE Table — Vitamin D**

| Reference | GRADE Certainty | Reason for GRADE Rating |
| --- | --- | --- |
| 20 | Moderate | Observational design; adequate sample; moderate risk of confounding. |
| 44 | Moderate | Small observational study; moderate risk of bias; limited adjustment. |
| 45 | Moderate | Large sample size improves confidence but cross-sectional design limits certainty. |
| 21 | Moderate | Case-control design; moderate bias; association plausible but confounding remains. |
| 22 | Moderate | Observational study with acceptable sample; moderate risk of confounding. |
| 23 | Moderate | Small sample; observational design; some methodological limitations. |
| 46 | Moderate | Retrospective design; risk of bias due to non-random allocation; large sample increases confidence. |
| 47 | Moderate | Case-control; moderate sample; potential selection and recall bias. |
| 48 | Moderate | Cross-sectional; moderate risk of confounding; limited adjustment. |
| 49 | Moderate | Case-control; acceptable sample; moderate risk of confounding. |
| 50 | Moderate | Case-control; moderate risk of confounding; inconsistent findings. |
| 24 | Moderate | Observational; moderate risk of bias; large sample slightly improves certainty. |
| 51 | Moderate | Case-control; some methodological limitations but adequate reporting. |
| 52 | Moderate | Large observational study; moderate confounding risk; acceptable outcome assessment. |
| 53 | Moderate | Cross-sectional; moderate bias; outcome measurement acceptable. |
| 54 | Moderate | Cross-sectional; small sample; moderate risk of bias; consistent direction of effect. |
| 55 | Moderate | Case-control; moderate risk of confounding; plausible biological mechanism. |
| 56 | Moderate | Case-control; moderate sample; methodological limitations present. |

**GRADE Table — Anaemia (Iron + Folic Acid)**

| Reference | GRADE Certainty | Reason for GRADE Rating |
| --- | --- | --- |
| 25 | Moderate | Case–control design; moderate sample size; moderate risk of bias and confounding. |
| 26 | Moderate | Large cross-sectional study; moderate risk of residual confounding. |
| 57 | Moderate | Large sample; observational design limits certainty; moderate risk of bias. |
| 58 | Moderate | Adequate sample; observational; confounders not fully controlled. |
| 59 | Moderate | Cohort study with strong association; downgraded for risk of confounding. |
| 60 | Moderate | Cross-sectional design; moderate sample; limited covariate adjustment. |
| 61 | Moderate | Large sample; observational design; moderate bias in outcome measurement. |
| 62 | Moderate | Moderate sample; cross-sectional; moderate risk of bias; fair outcome measurement. |
| 63 | Moderate | Observational design; moderate sample; incomplete confounder control. |
| 28 | Moderate | Large cross-sectional study; moderate residual confounding limits certainty. |

**GRADE Table — Multiple Micronutrient**

| Reference | GRADE Certainty | Reason for GRADE Rating |
| --- | --- | --- |
| 29 | Moderate | Cross-sectional design; moderate risk of confounding; adequate sample. |
| 31 | Moderate | Case-control design; moderate bias; reasonable sample size. |
| 64 | Moderate | Large cluster RCT but downgraded for implementation variability and contextual heterogeneity. |
| 65 | Moderate | Case-control; moderate risk of bias; adequate reporting; non-randomized. |
| 33 | Low | Cross-sectional; lacking key methodological details; high risk of residual confounding. |
| 34 | Moderate | Large case-control study; moderate risk of bias; consistent outcomes. |
| 35 | Moderate | Very large RCT but downgraded for inconsistency across outcomes and population variability. |
| 66 | Moderate | RCT; downgraded for variability in effect across outcomes; moderate risk of bias. |
| 30 | Moderate | RCT; downgraded for inconsistency in mortality outcomes and contextual differences. |
