# Supplementary material for: Impact of maternal micronutrient supplementation on pregnancy outcomes in developing countries: a systematic review and meta-analysis
Source: BMC Pregnancy Childbirth. 2026 May 13;26:731. doi: 10.1186/s12884-026-09210-1 (PMC13343903; doi:10.1186/s12884-026-09210-1)
Supplement: Supplementary file 4 — Supplementary Material 4 [file 12884_2026_9210_MOESM4_ESM.docx]

Table 1: Characteristics of Included Studies

(A) Zinc Studies (n = 12)

| **Ref.** | **Author** | **Country** | **Study Design** | **Sample Size** | **Population** | **Key Findings** |
| --- | --- | --- | --- | --- | --- | --- |
| 13 | Schulpis et al. | Greece | Case–control | 499 | Cord blood neonates | Zn deficiency associated with LBW |
| 20 | Nossier et al. | Egypt | Randomized trial | 675 | Zn-deficient pregnant women | Zn supplementation improved pregnancy outcomes |
| 14 | Jyotsna et al. | India | Prospective | 100 | Mother–newborn pairs | Zn significantly lower in LBW infants |
| 21 | Samimi et al. | Iran | Case–control | 129 | Mother–newborn pairs | No association with LBW |
| 22 | Badakhsh et al. | Iran | Cross-sectional | 140 | Mother–newborn pairs | Zn deficiency associated with LBW |
| 16 | Danesh et al. | Iran | Randomized trial | 110 | Mother–newborn pairs | No reported outcome |
| 6 | Iqbal et al. | Pakistan | Cross-sectional | 80 | Pregnant vs non-pregnant women | Zn deficiency linked to complications |
| 41 | Nanbakhsh & Tabrizi | Iran | Cross-sectional | 127 | Mother–newborn pairs | Lower Zn in LBW mothers |
| 42 | Bellad & K.S. | India | Cross-sectional | 200 | Pregnant women | Positive correlation with birth weight |
| 43 | Collins | Nigeria | Cross-sectional | 190 | Mother–newborn pairs | No significant association |
| 44 | Osendarp et al. | Bangladesh | Case–control | 559 | Mother–newborn pairs | No difference in birth weight with Zn supplementation |
| 45 | Karim et al. | Pakistan | Cross-sectional | 382 | Mother–newborn pairs | Maternal Zn consumption increased birth weight |

(B) Vitamin D Studies (n = 18)

| **Ref.** | **Author** | **Country** | **Study Design** | **Sample Size** | **Population** | **Key Findings** |
| --- | --- | --- | --- | --- | --- | --- |
| 19 | Ahmed et al. | Bangladesh | Cross-sectional | 515 | Pregnant women | 17.3% deficiency; no outcome reported |
| 46 | Salek et al. | Iran | Cross-sectional | 88 | Pregnant women | High vitamin D deficiency prevalence |
| 24 | Hantoosh et al. | Iraq | Cross-sectional | 500 | Pregnant women | 76% deficient |
| 25 | Kasim | Iraq | Case–control | 80 | Pregnant women | 95% of abortion cases deficient |
| 26 | Al-Rubaye et al. | Iraq | Cross-sectional | 88 | Mother–infant pairs | Strong maternal–neonatal correlation |
| 47 | Mohammed & Alqani | Iraq | Cross-sectional | 42 | Pregnant women | Vitamin D deficiency linked to early pregnancy loss |
| 27 | Oner Dirican & Korucu | Turkey | Retrospective | 2688 | Pregnant women | Supplementation lowered preterm & LBW |
| 28 | Mwafy et al. | Gaza | Case–control | 200 | Pregnant vs non-pregnant | Higher deficiency in pregnant women |
| 29 | Loudyi et al. | Morocco | Cross-sectional | 102 | Pregnant women | High deficiency; no effect on birth weight |
| 30 | Nasri et al. | Tunisia | Case–control | 132 | Pregnant women | Associated with neural tube defects |
| 31 | Charandabi et al. | Iran | Cross-sectional | — | Pregnant women | No association with delivery mode or birth weight |
| 48 | Gbadegesin et al. | Nigeria | Cross-sectional | 461 | Pregnant women | No association with pregnancy complications |
| 49 | Hossain et al. | Pakistan | Case–control | — | Pregnant women | Supplementation improved neonatal vit D & Apgar |
| 50 | Sabour et al. | Iran | Cross-sectional | 449 | Pregnant women | Supplementation improved Apgar & birth weight |
| 51 | Mehta et al. | Tanzania | Cross-sectional | 884 | Pregnant women | No LBW association; ↑ mother–child transmission risk |
| 52 | Toko et al. | Kenya | Cross-sectional | 63 | Pregnant women | Deficiency → 4× higher stunting risk |
| 53 | Dadwal & Narayan | India | Case–control | 100 | Pregnant women | Deficiency ↑ preterm birth risk |
| 54 | Sablok et al. | India | Case–control | 165 | Pregnant women | Supplementation ↓ SGA (non-significant vs IFA) |

(C) Anaemia / Iron–Folic Acid Studies (n = 10)

| **Ref** | **Author** | **Country** | **Study Design** | **Sample Size** | **Study Population** | **Key Findings** |
| --- | --- | --- | --- | --- | --- | --- |
|  | El-Farrash et al. | Egypt | Case–control | 80 | Mother–newborn pairs | LBW significantly higher in anaemic mothers |
|  | Finkelstein et al. | India | Cross-sectional | 366 | Pregnant women | 2× higher LBW in anaemic mothers |
|  | Bodeau-Livinec et al. | Benin | Cross-sectional | 1508 | Pregnant women | Severe anaemia ↑ LBW (PR 2.8) |
|  | Mahmood et al. | Pakistan | Cross-sectional | 622 | Pregnant women | Third-trimester anaemia → adverse outcomes |
|  | Nair et al. | India | Cohort | 1007 | Pregnant women | Anaemia ↑ LBW, SGA, perinatal death |
|  | Mahato & Shrestha | Nepal | Cross-sectional | 200 | Pregnant women | Severe anaemia → IUGR, LBW, perinatal death |
|  | Tunkyi & Moodley | South Africa | Cross-sectional | 2000 | Pregnant women | Higher stillbirth in anaemic group |
|  | Srour et al. | Palestine | Cross-sectional | 163 | Pregnant women | Anaemia associated with LBW & preterm |
|  | Suprapto et al. | Iraq | Cross-sectional | 500 | Pregnant women | IDA ↑ LBW, preterm, adverse outcomes |
|  | Bakhtiar et al. | Pakistan | Cross-sectional | 860 | Pregnant women | Anaemia → LBW, preterm, IUFD |

(D) Multiple Micronutrient Supplementation (MMS) (n = 9)

| **Ref** | **Author** | **Country** | **Study Design** | **Sample Size** | **Study Population** | **Key Findings** |
| --- | --- | --- | --- | --- | --- | --- |
|  | Elfane et al. | Morocco | Cross-sectional | 344 | Pregnant women | Higher MMN intake → normal birth weight |
|  | Fares et al. | Tunisia | Case–control | 907 | Pregnant women | Micronutrient deficiency ↑ LBW |
|  | West et al. | Bangladesh | Cluster RCT | 25,130 | Pregnant women | MMS ↓ LBW & preterm |
|  | Christian et al. | Nepal | Case–control | — | Pregnant women | MMS ↓ LBW vs control (no difference vs IFA) |
|  | Tukadoji et al. | India | Cross-sectional | — | Pregnant women | MMN intake positively correlated with birth weight |
|  | Bhutta et al. | Pakistan | Case–control | 2378 | Pregnant women | MMS ↓ LBW (not significant vs IFA) |
|  | Shankar et al. | Indonesia | Double-blind RCT | 31,290 | Pregnant women | MMS ↓ LBW by 18% |
|  | Fawzi et al. | Tanzania | Double-blind RCT | 8428 | Pregnant women | MMS ↓ LBW & SGA |
|  | Kæstel et al. | Guinea-Bissau | Randomized Trial | 2100 | Pregnant women | ↑ birth weight; no mortality benefit |
